# Microinjection of melanin-concentrating hormone (MCH) into the median raphe nucleus promotes REM sleep in rats

**DOI:** 10.5935/1984-0063.20200075

**Published:** 2021

**Authors:** Claudia Pascovich, Soﬁa Niño, Alejandra Mondino, Ximena Lopez-Hill, Jessika Urbanavicius, Jaime Monti, Patricia Lagos, Pablo Torterolo

**Affiliations:** 1 Facultad de Medicina, Universidad de la República, Fisiología, Montevideo - Uruguay.; 2 Instituto de Investigaciones Biológicas Clemente Estable, Neurofarmacología Experimental, Montevideo - Uruguay.; 3 Hospital de Clínicas, Farmacología y Terapéutica, Montevideo - Uruguay.

**Keywords:** Serotonin, Hypothalamus, Sleep, REM, Peptide, Paradoxical Sleep, Slow Wave Sleep

## Abstract

Melanin concentrating hormone (MCH) is a sleep-promoting neuromodulator synthesized by neurons located in the postero-lateral hypothalamus and incerto-hypothalamic area. MCHergic neurons have widespread projections including the serotonergic dorsal (DR) and median (MnR) raphe nuclei, both involved in the control of wakefulness and sleep. In the present study, we explored in rats the presence of the MCH receptor type 1 (MCHR-1) in serotonergic neurons of the MnR by double immunofluorescence. Additionally, we analyzed the effect on sleep of MCH microinjections into the MnR. We found that MCHR-1 protein was present in MnR serotonergic and non-serotonergic neurons. In this respect, the receptor was localized in the primary cilia of these neurons. Compared with saline, microinjections of MCH into the MnR induced a dose-related increase in REM sleep time, which was related to a rise in the number of REM sleep episodes, associated with a reduction in the time spent in W. No signiﬁcant changes were observed in non-REM (NREM) sleep time. Our data strongly suggest that MCH projections towards the MnR, acting through the MCHR-1 located in the primary cilia, promote REM sleep.

## INTRODUCTION

The melanin-concentrating hormone (MCH) is a neuropeptide synthesized by neurons whose cell bodies are located in the postero-lateral hypothalamus and incerto-hypothalamic area; these neurons project to various regions of the central nervous system (CNS)^1-5^. MCH exerts its biological function through two receptors, but only the MCH receptor type 1 (MCHR-1) is functional in rodents^6^.

MCHergic neurons are involved in physiological processes such as energy homeostasis, mood regulation and sleep^7-10^. A high density of MCHergic fibers and receptors has been demonstrated to be present in specific regions of the CNS, such as the dorsal (DR) and median (MnR) raphe nuclei, which are involved in the generation of wakefulness (W) and sleep^1,2,4,5,11-14^.

The serotonergic neurons of the mesopontine raphe nuclei play an important role in the control of REM sleep^12,15,16^. Previous studies have shown that microinjections of MCH into the DR produce a significant increase in REM sleep and a moderate increment in slow wave sleep (SWS)^17^. Moreover, microinjections of MCH into the DR promote a depressive-like behavior^9^. These effects were probably related to the fact that MCH decreases the firing rate of putative DR serotonergic neurons and the synaptic release of serotonin^11,18^.

Recent reports in rodents have described that the MCHR-1 is localized in neuronal primary cilia^14,19^. In this regards, Niño-Rivero et al. (2019)^19^ have shown that 4% of serotonergic and 12% of GABAergic DR neurons express the MCHR-1 in their primary cilia. With respect to the MnR, by means of intracerebroventricular (i.c.v.) microinjections of MCH conjugated with the fluorophore rhodamine (R-MCH), we demonstrated that serotonergic and GABAergic neurons of the MnR internalized R-MCH both in rats and in cats, suggesting that such neurons were MCH-receptive and probably express functional MCHR-1^13^. In addition, we also found that i.c.v. and juxtacellular administration of MCH decreases the firing rate of presumed serotonergic MnR neurons. Hence, the aims of the present study were: 1. to demonstrate the presence of MCHR-1 in MnR neurons, and to confirm if the receptor is located in their primary cilia; 2. to determine whether the receptor is present in serotonergic neurons; 3. to find out if, as in the DR, MCH microinjections into the MnR promote sleep.

## MATERIAL AND METHODS

### Animals

Nine male Wistar rats (250-310g) were used in this study. All the experimental procedures were conducted in accordance with the Guide for the Care and Use of Laboratory Animals (8^th^ edition, National Academy Press, Washington, DC, 2010) and the national law on animal experimentation (Law N° 18.611). The experimental protocols were approved by the School of Medicine Bioethics Committee (Protocols Nº 070153-00515-13 and Nº 070153- 000841-18). Adequate measures were taken to minimize pain, discomfort or stress of the animals, and all efforts were made to use the minimal number of animals necessary to produce reliable scientific data.

### Reagents and antibodies

The reagents to prepare the solutions were obtained from Sigma-Aldrich (Eugene, OR, USA). Biotinylated secondary antibodies, fluorophore-conjugated secondary antibodies and normal donkey serum (NDS) were obtained from Jackson ImmunoResearch (West Grove, PA, USA). Streptavidin conjugated with fluorophores were obtained from Molecular Probes- ThermoFischer Scientific (Eugene, OR, USA).

### Immunohistochemical procedures

The perfusion of three animals was performed during the light phase of the light/dark cycle. The animals were anaesthetized with urethane (1.5g/kg) and perfused with 0.9% NaCl followed by a 4% paraformaldehyde (PFA) solution. The brains were immediately dissected out and fixed by immersion in 4% PFA overnight. Thereafter, they were cryoprotected in 30% sucrose solution in 0.1M PB for 48h and frozen. Coronal sections (30µm) were obtained by a cryostat (Leica CM 1900, Leica Microsystems, Nussloch, Germany). Sections containing the MnR were between the levels AP -7.2-8.28mm (from Bregma) according to the atlas of Paxinos and Watson (2005)^20^. The sections were collected and stored in an anti-freeze solution at -20 °C until immunostaining procedures were performed.

Single and double immunofluorescence procedures were performed to detect MCHR-1 protein (goat anti-MCHR-1, sc-5534, Santa Cruz Biotechnologies; or rabbit anti-MCHR1, 702618, ThermoFisher Scientific, Il, USA; 1:1000) and serotonin (goat anti-serotonin, 20079, ImmunoStar, WI, USA; 1:500). Negative controls in all procedures consisted of omission of the primary antibodies. The identification of MCHR-1 in primary cilia and MCHR-1 in serotonergic neurons were described in Niño-Rivero et al. (2019)^19^.

### Histological data analyses

We examined six coronal sections per rat assayed by double immunofluorescence against MCHR-1 and serotonin obtained from three rats. In each coronal section, five 20x microphotographs were taken along the dorso-ventral axis of the serotonergic (central) region of the MnR, with a 20x objective lens on a Moticam Pro 282B camera (Motic, Hong Kong, China), coupled to an Eclipse 50i epifluorescence microscope (Nikon, Tokio, Japan). Photoshop and Image J softwares were used to quantify the number of serotonergic neurons in each microphotograph, as well as the number of MCHR-1 positive signals in structures recognized as primary cilia in our previous study^19^. The number of counted cells were averaged per animal; thereafter, a grand average was performed for all the animals. The values are presented as mean±S.E.M. (standard error of the mean).

### Surgical procedures for sleep recordings

Six rats were anesthetized with a mixture of ketamine-xylazine-acepromazine (90mg/kg, 5mg/kg and 2mg/kg, i.p., respectively), positioned in a stereotaxic frame (David Kopf Instruments, USA) and prepared for standard polysomnography. Following scalp incision, skull landmarks were visualized and screw electrodes (1mm diameter) were implanted in the skull for recording the frontal, parietal and occipital electroencephalogram (EEG); a referential electrode was implanted in the cerebellum. A bipolar electrode was inserted in the neck muscles to monitor the electromyogram (EMG). All the electrodes were soldered to a connector. In addition, a guide cannula (gauge 26) was inserted into the MnR (AP -7.8, L 2.6 and H 6.7mm from Bregma)^20^. Guide cannulas were lowered at an angle of 20º to avoid the sagittal vein and were placed 2mm above the MnR to minimize cellular damage at the injection site. The connector and cannula were cemented to the skull with dental acrylic. At the end of the surgical procedures, an analgesic (Ketoprofen, 1mg/kg, subcutaneously) was administered.

The animals were treated postoperatively for 24hs with antibiotics (Cefradine, 50mg/kg i.m.). A topical antibiotic cream (Neomycin) was applied to the skin margins surrounding the implant.

### Recording and microinjection procedures

After the surgery, the animals were maintained with food and water available *ad libitum* and kept under controlled conditions (temperature 22±2 °C, 12-h day/night cycle, lights on at 6:00 a.m.). Immediately after surgery, the animals were habituated to a soundproof chamber fitted with rotating connector during approximately five days. The endpoints of the adaptation recording sessions were determined when the animals showed consistent REM sleep time and latencies for at least three consecutive recording sessions. Bioelectric signals were amplified (X1000), filtered (0.5-500Hz), sampled (512Hz, 16 bits) and stored in a PC using Spike 2 software (Cambridge Electronic Design, Cambridge, UK).

MCH (50 and 100ng in 0.2µl of sterile saline, Phoenix Pharmaceutical Inc., Belmont, CA) or vehicle (0.2µl of sterile saline) was microinjected into the MnR during a period of 2 minutes, with an injection cannula (28 gauge), which extended 2mm beyond the guide cannula. Aliquots for the doses employed were prepared and frozen at -20 °C, and thawed immediately before use. Microinjections were always performed during the dark phase at approximately 6.00 p.m. Recording sessions began 5min later and lasted for 6h. Each animal received three microinjections (vehicle, 50 and 100 ng of MCH); only one microinjection was performed during each recording session and a minimum of 2 days were left between each one. A balanced order of MCH and control microinjections was used to merge the effects of both the drug and the time elapsed during the experimental protocol.

Chinese black dye (0.2ml) was microinjected at the end of the experiments in order to identify the microinjection site. The rats were euthanized with an overdose of urethane and perfused with 4% PFA. The brains were removed, cryoprotected in a solution of sucrose 30% and cut in 30µm coronal sections with a cryostat. Selected sections were stained with Nissl.

### Sleep scoring and data analysis

Data were acquired and processed by Spike 2 software. The polysomnographic data was visually scored in 10 second epochs; the predominant activity of each epoch was assigned to the following categories based in standard criteria: W, light sleep (LS), SWS, REM sleep and non- REM (NREM) sleep (LS+SWS). Latencies to SWS (from the beginning of the recording to the first of two consecutive epochs of SWS) and REM sleep (from the beginning of the recording to REM sleep onset), as well as the number and mean duration of W and sleep episodes were also determined.

All values are presented as mean±S.E.M. (standard error of the mean). The statistical significance of the difference between controls vs. MCH effects was evaluated using one way analysis of variance (ANOVA) and Dunnett post hoc test (one-tailed analysis). The criterion used to discard the null hypothesis was *p*<0.05.

## RESULTS

**Figure 1:**
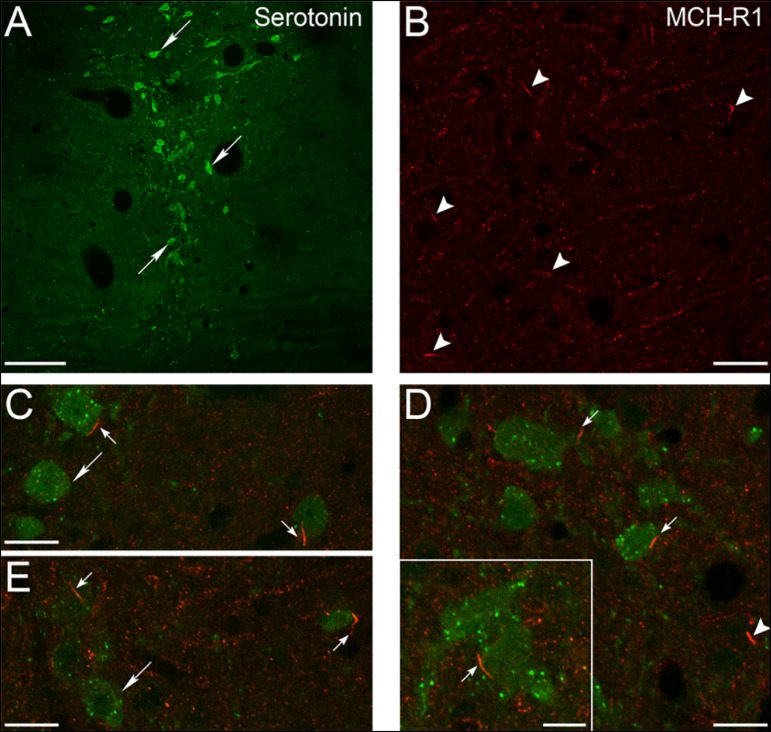
Localization of MCHR-1 in neuronal primary cilia within the MnR. A-B. Topographic microphotographs of the MnR showing positive immunofluorescence signals for serotonin (big arrows in A) and MCHR-1 (arrowheads in B), respectively. C-E. Microphotographs showing several examples of double immunofluorescence assays. MCHR-1 located in the primary cilia is present in serotonergic somata (small arrows). Serotonergic neurons without MCHR-1 are indicated by big arrows, and the presence of MCHR-1 in non-serotonergic neurons is indicated by arrowheads. The inset in D shows a higher magnification a MCHR-1 labeled primary cilia present in a serotonergic neuron. Calibration bars: A, 100 µm; B, C, D and E, 20 µm; D, 10 µm.

Figure 1A shows a topographic view of the location of the serotonergic neurons within the MnR. Figure 1B shows a representative image of the MCHR-1 immunoreactivity (IR) in this nucleus; it is readily observed disperse punctuate MCHR-1-IR and rod-like immunoreactive structures (arrowheads). In a recent study, we identified these rod-like structures as primary cilia by the presence of type III adenylyl cyclase (AC-III), a well-known neuronal ciliary marker^19^. By double immunofluorescence, we were able to identify the presence of MCHR-1 as an appendage protruding from the somata of serotonergic neurons and non-serotonergic neurons (Figures 1C, 1D e 1E). In this respect, we counted 38.7±4.6 serotonergic neurons and 17.1±2.5 primary cilia expressing the MCHR-1; MCHR-1 was recognized in 2.9% of the serotonergic neurons. Thus, the neurochemical phenotype of most MnR neurons that present MCHR-1-IR remains to be determined.

**Figure 2:**
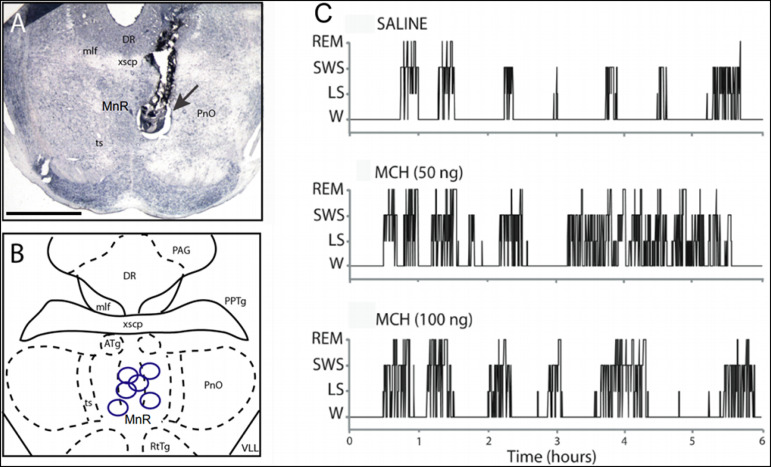
Microinjections of MCH were localized in the median raphe nucleus. A. Photomicrograph of a brainstem coronal section of a representative rat at the level of the MnR. The microinjection site is recognizable by the cannula track and a black dye deposit (arrow). The coronal section was stained with Nissl. Calibration bar, 2 mm. B. Schematic drawing showing the location of the microinjection sites. Each spot corresponds approximately to the center of the cannula tip, and its position was adjusted to the prototypical coronal section (AP -7.8 mm from Bregma) of Paxinos and Watson (2005). Abbreviations: ATg, anterior tegmental nucleus; DR, dorsal raphe nucleus; mlf, medial longitudinal fascicule; MnR, median raphe nucleus; PAG, periaqueductal grey; PnO, pontine reticular oral; PPTg, pedunculopontine tegmentum; RtTg, reticulotegmental; ts, tectospinal tract; VLL, ventral nucleus of the lateral lemniscus; xscp, superior cerebellar peduncle. C. Representative hypnograms illustrating the occurrence of wakefulness and sleep following microinjection of MCH into the MnR of a representative rat. The effects of the vehicle (saline), 50 ng, and 100 ng of MCH are exhibited.

MCH (50 and 100ng) and vehicle (saline) were microinjected into the MnR in six rats. In all of them, the tips of the cannulas were localized within the MnR. A photomicrograph showing an example of the cannula track and the dye deposit in the target site is shown in Figure 2A; a scheme of the approximate locations of the microinjections sites is displayed in Figure 2B.

**Figure 3:**
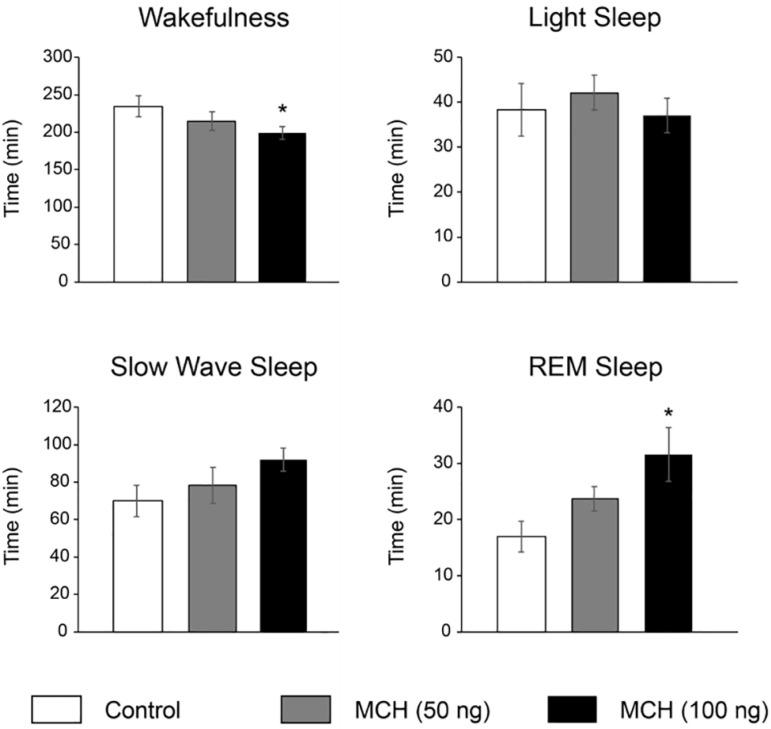
Effects on sleep and wakefulness of MCH microinjections into the median raphe nucleus. Bar charts show the time spent in wakefulness, light sleep, slow wave sleep and REM sleep. *, p < 0.05 compared with vehicle microinjections (ANOVA and Dunnett Multiple Comparisons test).

Figure 2C shows the effects on sleep of the microinjections of saline and MCH in a representative animal. In this case, it is readily observed in the hypnograms that both doses of MCH increase sleep time. The results obtained for all the treated animals are summarized in Table 1 and Figure 3. MCH 100ng increased REM sleep time from a control value of 16.9±2.8 min (4.7% of the total recording time) to 31.6±4.8 min (8.7 % of the total recording time).

**Table 1.** Effects on sleep of MCH microinjections into the median raphe nucleus

Wakefulness	Saline (1)	MCH (50ng) (2)	MCH (100ng) (3)	F	p (ANOVA)	p (2 Vs. 1)	p (3 Vs. 1)
Time (min)	234.7 ± 14.2	214.8 ± 12.3	199.3 ± 8.8 *	4.19	**0.03**	0.07	**0.01**
Number of episodes	80.5 ± 11.0	86.0 ± 9.3	91.0 ± 9.6	0.60	0.56		
Episode duration (min)	3.1 ± 0.4	2.8 ± 0.5	2.3 ± 0.2	2.40	0.12		
**Light sleep**							
Time (min)	38.3 ± 5.9	42.0 ± 4.0	37.0 ± 3.9	0.37	0.67		
Number of episodes	142.0 ± 16.9	151.8 ± 18.2	150.0 ± 14.9	0.59	0.56		
Episode duration (min)	0.3 ± 0.01	0.3 ± 0.01	0.3 ± 0.01	1.93	0.17		
**Slow wave sleep**							
Time (min)	69.9 ± 8.3	78.2 ± 9.5	92.0 ± 6.2	2.89	0.08		
Number of episodes	108.8 ± 11.3	122.3 ± 17.7	123.2 ± 11.1	1.24	0.31		
Episode duration (min)	0.7 ± 0.07	0.7 ± 0.03	0.8 ± 0.07	1.30	0.29		
**NREM sleep (LS + SWS) **							
Time (min)	649.6 ± 71.2	760.5 ± 65.8	806.5 ± 10.3	2.19	0.14		
**REM sleep**							
Time (min)	16.9 ± 2.8	23.6 ± 2.1	31.6 ± 4.8 *	4.09	**0.03**	0.14	**0.01**
Number of episodes	16.3 ± 1.6	24.0 ± 3.8 *	26.0 ± 2.7 *	5.50	**0.01**	**0.01**	**0.01**
Episode duration (min)	1.1 ± 0.1	1.1 ± 0.2	1.2 ± 0.1	0.79	0.47		
**Latency**							
REM sleep latency (min)	224.7 ± 65.6	223.0 ± 71.0	181.6 ± 47.2	1.42	0.27		
SWS latency (min)	420.8 ± 57.7	400.5 ± 82.9	280.0 ± 57.9	0.77	0.47		

* p<0.05; significant statistical difference with respect to control, ANOVA and Dunnett Multiple Comparisons test

REM sleep increase was due to a greater number of REM sleep episodes. In contrast, no effect was observed either in the average duration of the REM sleep episodes or REM sleep latency.

MCH 100ng did not affect either LS, SWS or NREM sleep parameters. However, MCH tended to increase SWS time (*p*=0.08). Compared to saline, MCH 100ng reduced the time spent in W (Table 1 and Figure 3).

No significant changes on W and sleep parameters were obtained after the administration of the lower dose of MCH (50ng), except for an increase in the number of REM sleep episodes (Table 1).

**Figure 4:**
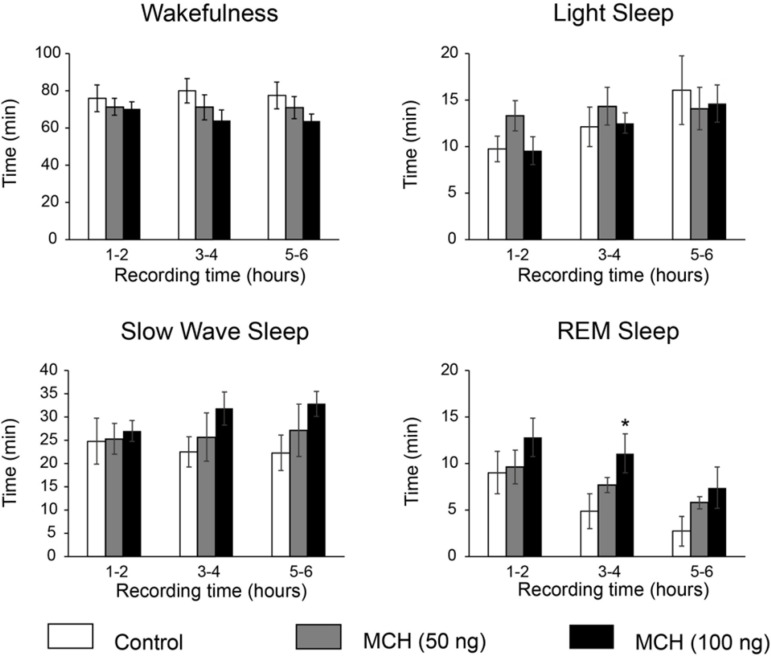
Effects on sleep and wakefulness of MCH microinjections into the median raphe nucleus. Bar charts show the time spent in wakefulness, light sleep, slow wave sleep and REM sleep analyzed in 2-hours blocks. *, p < 0.05 compared with vehicle injection (ANOVA and Dunnett Multiple Comparisons test).

The effects of MCH on W and sleep were also analyzed in two-hour blocks (Figure 4). Although MCH tended to increase REM and SWS in all the blocks, the significant effects were restricted to the second two-hours block, where MCH 100ng produced a significant increase in REM sleep.

## DISCUSSION

In the present study, by means of immunohistochemistry we demonstrated the presence of MCHR-1 within the MnR. MCHR-1-IR was identified in rod-like structures that were previously recognized as primary cilia^14,19^. We were able to identify that serotonergic neurons possess MCHR-1-labeled primary cilia; MCHR-1 was also recognized in primary cilia of unidentified MnR neurons. In addition, microinjections of MCH into the MnR produced a dose- dependent increase of the time spent in REM sleep, which was related with an increase in the number of episodes and associated with a reduction of the time spent in W.

### MCHR-1 is present at primary cilia of the MnR neurons

We have previously demonstrated that MCH-containing fibers are present in the MnR, where they are in close apposition to serotonergic and non-serotonergic neurons^13^. In addition, we have determined that R-MCH is internalized in both serotonergic and GABAergic neurons of the MnR, suggesting that MCHR-1 is present in these neurons. In fact, it is well-known that when a neuropeptide interacts with its receptor, the neuropeptide-receptor complex is usually internalized^11,21^.

The presence of MCHR-1 in MnR neurons was now confirmed by immunohistochemistry against this protein. Niño-Rivero et al. (2019)^19^ showed by AC-III immunohistochemistry in the adjacent DR, that the primary cilia exhibit a rod-like aspect, and that MCH-R1 is localized in these appendages. In the present study, we also demonstrated that MnR serotonergic neurons possess MCHR-1-IR in the same type of structures; i.e., in their primary cilia. This fact does not exclude the possibility that the MCH-R1 is also present at other neuronal sites, but is likely more concentrated at these appendages.

Serotonergic neurons are located mainly along the midline or central region of the MnR^22,23^. According to Jacobs and Azmitia (1992)^24^ and Vertes and Crane (1997)^x^, the MnR of the rat contains approximately 1,110 and 4,114 serotonergic neurons, respectively^24^. We were able to determine that 2.9% serotonergic neurons possess MCHR-1-IR. This small percentage is likely because the antigen retrieval method reduces serotonin immunoreactivity, and thus decreases the number of identified serotonergic neurons^19^. Furthermore, the primary cilia are small (approximately 10µm long) and serotonin and MCHR-1 do not co-localize in the same cellular compartment (i.e., serotonin is not present in the primary cilia). Hence, although it is not possible to see a clear cellular continuity between the MCHR-1-labelled primary cilia and the corresponding neuronal body with our method, the very close apposition observed in the same plane strongly suggest continuity^19^. In spite of the preceding arguments, the percentage of serotonergic neurons in which we were able to recognize MCH-R1 in their primary cilia was similar in the DR^19^ and MnR (≈4 and 2.9%, respectively). The fact that the effect of MCH on putative serotonergic neuronal firing is overwhelming (see below), strongly suggest that the presence of the MCH-R1 in the serotonergic neurons is underestimated by the above-mentioned technical issues.

The MnR also contains GABAergic and glutamatergic neurons that are located both in the central and paracentral (lateral) region of the nucleus^22,23^. In fact, previous data suggest that GABAergic neurons within the MnR express the MCHR-1^13^. Hence, as in the work of Niño-Rivero (2019)^19^, in pilot experiments we tried to identify GABAergic neurons by mean of GAD- 67 immunohistochemistry. However, because GAD-67 tends to accumulate in axon terminals and not in the somata, we were not able to label a considerable number of GABAergic neurons within the MnR. The administration of a substance that blocks axonal transport (i.e., colchicine) is required to reveal the entire population of GAD-67 immunoreactive neurons^25^. Although we carried out this approach, this treatment disrupted MCH-R1 immunoreactivity.

### MCH microinjections into the median raphe promotes REM sleep

Intracerebral microinjection of MCH is a technical approach in which our group has extensive experience^26-28^. Although we verified that the cannula tips were confined within the limits of the MnR, it is not possible to exclude the diffusion of MCH outside this nucleus. The diffusion rate of a given drug depends upon a number of factors including its diffusion coefficient^29^; but as a guide, it is known that methylene blue microinjected into the CNS in a 0.2µl volume diffuses an average ratio of 520µm^30^. Because of the small doses and volume employed in the present study, we consider that the diffusion of effective concentrations of MCH outside the MnR, if present, was negligible. In fact, our group has demonstrated that microinjections of MCH into the nearby sublaterodorsal tegmental nucleus of the rat produces the opposite effect, i.e., a decrease in REM sleep time^28^.

MCH produced a dose-dependent increase in REM sleep. Previous studies have demonstrated that DR and MnR serotonergic neurons have a REM-off firing profile^24^. In addition, acute inactivation of the DR serotonergic neurons by cooling or by microinjection of 5HT1-A or GABA-A agonists increases the time spent in REM sleep^31-34^. According to these data and the classical reciprocal interaction model of REM sleep generation^35,36^, it has been proposed that these neurons play a permissive role in the generation of REM sleep. In other words, their firing rate should be decreased in order to generate REM sleep. Of note, Pascovich et al. (2020)^13^ determined that i.c.v. administration of MCH resulted in a significant decrease in the firing rate of 53% of MnR neurons, while juxtacellular administration of MCH reduced the firing rate in 67% of these neurons. Also, juxtacellular administration of the MCHR-1 antagonist (ATC-0175) produced the opposite effect. Taking into account that some of these neurons were putative serotonergic according to their electrophysiological pattern of discharge, it was proposed that MCH inhibits serotonergic MnR neurons, and in conjunction with the inhibition of DR serotonergic neurons, this inhibition promotes REM sleep.

The effects on sleep of MCH microinjections into the DR during the light phase were explored by Lagos et al. (2009)^17^. As in the present study, they observed minimal effect on sleep with MCH 50ng. Administration of MCH 100ng into the DR induced an increase of REM sleep and SWS, while the effect in the MnR (the present study) was limited to an increment in REM sleep values. Additionally, the effect on REM sleep in the DR was observed in the three 2-hours blocks, while at the level of the MnR it was limited to the second 2-hours block. These results are in accordance with the fact that MCH suppresses the activity of a greater percentage of putative serotonergic neurons within the DR (80%) than in the MnR (57%)^11,13^. Overall, these data suggest that the effects of MCH on the DR are likely more powerful than on the MnR.

MCHergic neurons increase their firing rate on passing from W to NREM sleep, and reach the maximal firing rate during REM sleep^37^; REM sleep active MCHergic neurons were also identified by microendoscopy in genetically-modified mice^38^. Moreover, i.c.v. administration of MCH to the rat produces a marked increase in REM sleep and a moderate increase in NREM sleep^39^, whereas the systemic administration of a MCHR-1 antagonist produces the opposite effect^40^. Optogenetic and chemogenetic methodologies also showed that MCH promotes sleep, mainly REM sleep^41-43^. Hence, different experimental findings strongly suggest that MCH is a powerful hypnogenic factor that plays a crucial role in the control of sleep, mainly REM sleep^44,45^. It can be considered that the REM sleep-promoting effect of MCH applied into the MnR (present study) is an additional component of the REM sleep promoting action of the MCHergic neurons projecting towards the DR^17^, *locus coeruleus*^27^, basal forebrain^46^, and probably other regions of the brain^47^.

### Translational implications

A decrease in REM sleep latency, as well as an increase in REM sleep duration and the amount of eye movements in each REM sleep episode, have been considered biological markers of depression, which might predict its relapse and recurrence^48^. MCH may play a role in depression^9,49^. In this regards, Lopez-Hill et al. (2013)^50^ have shown that 100ng of MCH (but not 50ng) applied into the MnR significantly increased the immobility time and decreased the swimming time in the forced-swimming test, thus demonstrating a depressive-like effect. Similar outcomes were observed when MCH was applied into the DR^51^. Hence, as shown previously with respect to the DR, MCH microinjected into the MnR induces REM sleep and a depressive- like effect, which tends to suggest that MCH projections towards the MnR contribute to the occurrence of mood disorders.

## CONCLUSION

The present study demonstrate that MCHR-1 is present in the primary cilia of MnR serotonergic and non-serotonergic neurons. It was also shown that the modulation of the MnR neuronal activity by MCH promotes the generation of REM sleep. MCHergic neurons may act in tandem in the DR and MnR (as well as in other nuclei) in order to induce this behavioral state.
